# Characterization of a Dimeric Arginase From *Zymomonas mobilis* ZM4

**DOI:** 10.3389/fmicb.2019.02755

**Published:** 2019-11-26

**Authors:** Seung-A Hwangbo, Ji-Won Kim, Sun-Ju Jung, Kyeong Sik Jin, Jie-Oh Lee, Jeong-Sun Kim, Suk-Youl Park

**Affiliations:** ^1^Pohang Accelerator Laboratory, Pohang University of Science and Technology, Pohang, South Korea; ^2^Institute of Membrane Proteins, Pohang University of Science and Technology, Pohang, South Korea; ^3^Department of Chemistry, Chonnam National University, Gwangju, South Korea

**Keywords:** arginine, arginase, ARG, dimer, structure, GGDCS motif

## Abstract

Many organisms have genes to protect themselves from toxic conditions such as high ethanol and/or ammonia concentrations. When a high ethanol condition is induced to *Zymomonas mobilis* ZM4, a representative ethanologenic organism, this bacterium overexpresses several genes to overcome this ethanol stress. Among them, we characterized a gene product annotated as an arginase (zmARG) from *Z. mobilis* ZM4. Even though all of the arginase-determining sequence motifs are not strictly conserved in zmARG, this enzyme converts L-arginine to urea and L-ornithine in the presence of a divalent manganese ion. The revealed high-resolution crystal structure of zmARG shows that it has a typical globular α/β arginase fold with a protruded C-terminal helix. Two zinc ions reside in the active site, where one metal ion is penta-coordinated and the other has six ligands, discerning this zmARG from the reported arginases with two hexa-liganded metal ions. zmARG forms a dimeric structure in solution as well as in the crystalline state. The dimeric assembly of zmARG is formed mainly by interaction formed between the C-terminal α-helix of one molecule and the α/β hydrolase fold of another molecule. The presented findings demonstrate the first reported dimeric arginase formed by the C-terminal tail and has two metal ions coordinated by different number of ligands.

## Introduction

Arginine catabolism generates two metabolic intermediates, L-ornithine and urea. L-ornithine is further converted into citrulline through the catalytic activity of transcarbamoylase in the urea cycle (also known as the ornithine cycle) that removes and excrete the highly toxic ammonia outside the organism. Arginase (Arginine amidinase; EC 3.5.3.1) is one of the enzymes involved in the final step of the urea cycle (Krebs and Henseleit, 1932). A deficiency of the arginase activity in human results in Argininemia, a disease caused by accumulation of arginine and ammonia in the blood, resulting in the development of neurological problems and other symptoms (Wong et al., 1993). Known arginases commonly have a typical α/β hydrolase fold and a conserved two metal ion-binding sequence (Kanyo et al., 1996). Therefore, they are speculated to be an ancestor enzyme of histone deacetylases and polyamine deacetylases (Hai et al., 2017).

Arginases frequently utilize dual manganese ions and a water molecule for a nucleophilic attack. The metal-associated hydroxide ion attacks the guanidine group of arginine, which results in cleavage of the covalent bond between the N^ε^ and C^ζ^ atoms of the arginine (Kanyo et al., 1996). In the reported arginase structures, their active sites contain a binuclear manganese cluster that is coordinated by one or two water molecules and two histidine and four aspartate residues from three conserved sequence motifs of GGDHS, DAHXD, and SXDXDXDP (Kanyo et al., 1996; Bewley et al., 1999). In the mechanism proposed by Christianson (2005), the hydroxide ion generated from the deprotonated water molecule by two manganese ions forms a tetrahedral intermediate with the C^ζ^ atom of the amine group of arginine. Then, the proton transfers by the other histidine and aspartate residues separate the amine group to give the ultimate products of the urea cycle, L-ornithine and urea (Kanyo et al., 1996).

The representative ethanologenic bacteria *Zymomonas mobilis* ZM4 over-expresses several genes under the high alcohol-stressed condition (Yang et al., 2013). Among them, the ZMO0432 gene product annotated as an arginase (zmARG) has the three sequence motifs found in arginase proteins. However, it shares a low sequence similarity to the previously reported arginases. In addition, the first metal-coordinating histidine residue of the GGDHS motif at the active site is replaced with cysteine in zmARG, indicating that the molecular interaction between the substrate and the enzyme slightly differs from the previously suggested binding mode in other arginases. Furthermore, the other two conserved sequence motifs (DAHXD and SXDXDXDP) are not strictly conserved, raising the question whether zmARG possesses an arginase activity or not. In order to provide a molecular background for the putative zmARG protein, we characterized its catalytic activity. The elucidated crystal structure of zmARG shows a common arginase fold with a protruding α-helix at the C-terminus that mediates a dimerization of the protein. The revealed biochemical property and the structural relationship with other arginases are discussed.

## Materials and Methods

### Cloning, Expression, and Purification of zmARG

The ZMO0432 gene encoding zmARG (Met1∼Lys290) was amplified from *Z. mobilis* subsp. *mobilis* ZM4 genomic DNA by polymerase chain reaction (PCR) using the two primers of 5′-CGATACCATATGAGTAGTATTAATAAACCGTTGAGACTC ATTTTCCCG-3′ and 5′-CGTCTCGAGTTATTTCCCGATTAA AGGCAGCTCTTCGAG-3′, which contains the *Nde*I and *Xho*I restriction sites (underlined), respectively. The amplified PCR product was treated with the restriction enzymes *Nco*I (New England Biolabs, Beverley, MA, United States) and *Xho*I (New England Biolabs, Beverley, MA, United States) and was inserted into the pSKB3 bacterial expression vector that expresses 25 extra residues at the N-terminus including a cleavable six-histidine residues followed by the tobacco etch virus (TEV) protease cleavage site. The constructed recombinant plasmid was transformed into *Escherichia coli* BL21^∗^(DE3) Star that was grown in LB medium or in a seleno-L-methionine (Se-Met) based medium in B834 (DE3) containing 100 μg⋅ml^–1^ ampicillin at 310 K. When the optical density at 600 nm reached 0.5, the fusion protein was expressed by adding 1.0 mM Isopropyl β-D-1-thiogalactopyranoside (IPTG) into the culture media followed by incubation for an additional 8 h at 310 K. The culture was harvested by centrifugation at 5,000 *g* at 277 K. The cell pellet was resuspended in an ice-cold buffer A (20 mM Tris–HCl (pH 7.5) and 150 mM NaCl) and disrupted by ultrasonication. The cell debris was removed by centrifugation at 11,000 *g* for 1 h. The expressed zmARG fusion protein was initially bound by a 5 ml HisTrapHP chelating column (GE Healthcare, Uppsala, Sweden) and the bound protein was eluted by a 500 mM imidazole gradient in buffer A. The eluted zmARG protein was incubated with the recombinant TEV protease and simultaneously dialyzed to remove the salt. After dialysis, the added TEV protein was removed by reloading the protein on the 5 ml HisTrap HP chelating column. The protein containing two additional amino acids (GH) at the N-terminus was further purified by gel filtration on a Superdex 200 column (GE Healthcare, Uppsala, Sweden) under the same buffer condition. For phasing, Se-Met substituted zmARG was prepared in a similar manner to the native protein.

### Arginase Activity Assay

The initially purified 31 μM zmARG protein by Ni-NTA affinity chromatography was used to measure for catalytic activity and dialyzed with the buffer A with 1 mM Ethylenediaminetetraacetic acid (EDTA) to remove metal ions. Generic arginase inhibitor, 2(S)-amino-6-boronohexanoic acid (ABH), was added to the enzyme at final concentration of 5 mM before reactions were initiated by the addition of arginine substrate (Baggio et al., 1999). Arginase activity was measured with arginase activity assay kit (Sigma-Aldrich, United States), according to the manufacturer’s protocol. The produced urea reacts to generate a colored product that was measured at 450 nm using an absorbance microplate reader (TECAN infinite 200, TECAN Deutschland GmbH, Crailsheim, Germany). The arginase activity of each sample was calculated considering the absorbance of a standard solution.

### Crystallization, Data Collection, and Structure Determination

The purified recombinant zmARG protein was concentrated to 10 mg⋅ml^–1^ in buffer A. The protein concentration of the purified zmARG was determined from the absorbance at 280 nm using its extinction coefficient (1.308 M^–1^⋅cm^–1^). Initial crystallization conditions were screened with the sparse-matrix method (Jancarik and Kim, 1991) using commercially available crystallization kits from Hampton Research (Aliso Viejo, CA, United States) and Rigaku Reagents (Bainbridge Island, WA, United States). The protein solution (1 μl) at a concentration of 10 mg⋅ml^–1^ was mixed with equal volumes of the screening reagents and the resulting drops were equilibrated against 70 μl reservoir solution at 295 K. The initial conditions were optimized with the hanging-drop vapor-diffusion method using the VDX plate (Hampton Research). The best crystals were grown in the presence of 25% (w/v) Polyethylene glycol 3350, 0.2 M Lithium Sulfate, and 0.1 M Tris–HCl (pH 8.5) at 295K within 3 days. For data collection, crystals were briefly immersed into a precipitant solution containing 25% (v/v) Ethylene glycol as a cryo-protectant and immediately placed in a 100 K nitrogen-gas stream. Native data sets of the zmARG crystals and a single-wavelength anomalous dispersion (SAD) data for SeMet-substituted zmARG crystals were collected at the 11C Micro-MX beamline of the Pohang Accelerator Laboratory (PAL) at a wavelength of 0.9795 Å, respectively. The data were then indexed, integrated, and scaled with *HKL2000* suite (Otwinowski and Minor, 1997). The crystals belong to the *C*2 space group with unit-cell parameters, *a* = 160.3, *b* = 100.5, *c* = 79.6 Å, α = γ = 90, and β = 114.5°. Ten out of the expected 16 selenium sites in the asymmetric unit were identified from the SAD data collected at a resolution of 2.5 Å using the program SOLVE (Terwilliger, 2003). The calculated electron density was improved by density modification using the program RESOLVE (Terwilliger, 2003) and PHENIX (Adams et al., 2010), resulting in automated modeling of approximately 80% of the residues. Further model building was performed manually using the program *Coot* (Emsley and Cowtan, 2004) and subsequent refinement was performed with PHENIX (Adams et al., 2010).

### Dynamic Light Scattering

The particle size and size distribution of the purified recombinant zmARG protein was determined with the help of Malvern Zetasizer Nano ZS90 (Malvern Instrument, United Kingdom), equipped with a He-Ne laser (max. 4 mW) operating at the wavelength of 633 nm at 25°C.

### Asymmetrical Flow Field-Flow Fractionation With Multi-Angle Light Scattering (AF4-MALS)

The molecular weight of the purified recombinant zmARG protein was determined by AF4-MALS detector (Wyatt, IN, United States) using an Eclipse DUALTEC AF4 system equipped with a standard channel (25 cm), 350 μm spacer and regenerated cellulose (10 kDa cutoff) membrane. Detection was accomplished with a DAWN Heleos II 18-angle MALS. Proteins were injected into a detector at a flow rate of 0.6 ml/min and a cross flow rate of 2.5 ml/min, respectively. Data were analyzed using the Zimm model for fitting experimental light scattering data and graphed using EASI graph with a RI peak in ASTRA 6.1 software (Wyatt, IN, United States).

### Accession Codes

The atomic coordinates and structure factors were deposited in the Protein Data Bank using an accession code 6KSY.

## Results

### Enzyme Activity of zmARG

**FIGURE 1:**
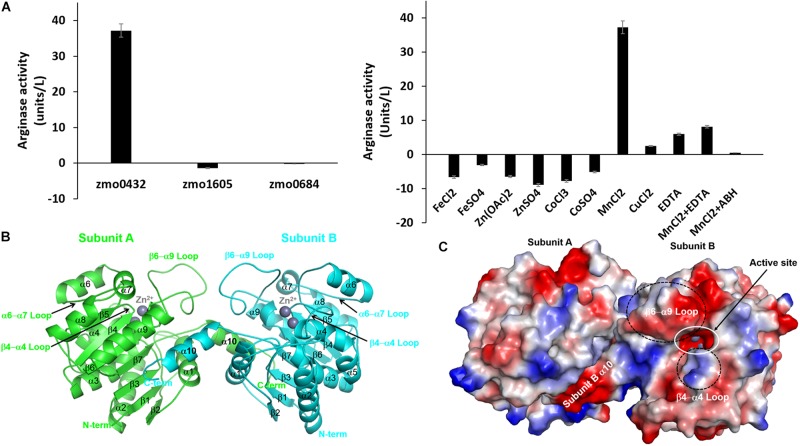
Characterization of the recombinant zmARG. **(A)** The arginase activity of recombinant ZMO0423 (zmARG). Left: Comparison of the arginase activity obtained in endpoint assays of the recombinant ZMO0423 protein, the recombinant ZMO1605 (pyruvate dehydrogenase E1 component beta subunit), and the ZMO0684 protein (CRISPR-associated protein Csy3 family). The arginase activity of each sample was calculated using the absorbance measured at 450 nm. Right: The arginase activity of zmARG in the presence of various metal ions or EDTA of 1.0 mM. 3 μM of the zmARG protein was used for assays. **(B)** Ribbon diagram of the dimeric zmARG structure. Each subunit of the dimeric protein is colored by green and cyan, respectively. Zinc ions are represented by gray balls. Figures were prepared using the *PyMOL* molecular-graphics program (Schrödinger, LLC). **(C)** Electrostatic surface representation of the zmARG dimer prepared with *PyMOL*. Zinc ions are drawn by gray ball.

We selected a gene annotated as an arginase (zmo0432) and two referenced genes of a pyruvate dehydrogenase E1 component beta subunit (zmo1605) and a CRISPR-associated protein Csy3 family (zmo0684) and made their expression constructs for *E. coli.* For the arginase activity of the purified recombinant proteins, 0.2 mg of each protein was mixed with 7.0 mg of the substrate. Among the tested proteins, only the zmo0432 gene product (zmARG) shows significant enzymatic activity against the used arginine substrate (left in Figure 1A). In order to assess its metal dependency, we also tested the catalytic activity of the enzyme in the presence of diverse divalent and trivalent metal ions including copper, manganese, cobalt (II), cobalt (III), iron or zinc. Among them, an arginase activity of zmARG was observed only with manganese ions. Notably, the presence of the divalent metal chelator EDTA reduced its enzyme activity by ∼ 85%, suggesting necessity of the metal ion for catalysis of arginine degradation by zmARG (right in Figure 1A).

The subsequent assays revealed that zmARG follows the typical Michaelis–Meten behavior with the kinetic parameters of 6.8 mM of Km and 302 s^–1^ of Kcat (Supplementary Figure S1) without the sigmoidal curve of the reaction products, suggesting no cooperativity of the enzyme for its catalytic activity. To assess an effect of zmARG to common arginase inhibitor, substrate analogue 2(S)-amino-6-boronohexanoic acid (ABH) was tested at a concentration of 5 mM in competitive inhibition assays against arginine as the substrate (Supplementary Figure S1). Interestingly, the ABH led to significant reduction in enzymatic activity by ∼95%, suggesting high susceptibility to ABH. The kinetic constants for zmARG and the previously reported values of hsARG, rnARG and bcARG (Lavulo et al., 2002; Alarcon et al., 2006; Garcia et al., 2015) are summarized in Supplementary Table S1.

### Overall Structure of zmArg

In order to get the structural background for the enzymatic activity of zmARG, its crystal structure was determined at a resolution of 1.65 Å. We have tried to get the phase information of the crystal through molecular replacement using the structures of reported arginases as a search model, but it was not successful. Therefore, the seleno-L-methionine-derivatized protein crystal was prepared and analysis of the SAD data gave an interpretable electron density.

The refined structure shows four molecules in the asymmetric unit of the C2 space group. Four molecules are almost identical as indicated from the small the root-mean-square deviation (RMSD) values of less than 0.4 Å for all the superposed Cα atoms. Noticeable deviations are found at both the extreme N-termini and the C-termini. Five residues at the extreme N-terminal region (Met1-Asn5) have weak electron densities. Geometry analysis of the modeled residues by WinCoot (Emsley and Cowtan, 2004) and Molprobity (Davis et al., 2004; Chen et al., 2010) reveals that all the modeled residues are located in valid regions of the Ramachandran plot (Table 1).

**TABLE 1:** Data collection and refinement statistics.

**Parameters**	**High resolution**	**Se-peak**
Synchrotron	11C Micro-MX, PAL	11C Micro-MX, PAL
Wavelength (Å)	0.97942	0.97942
Space group	C2	C2
Cell parameters	*a* = 160.1, *b* = 100.3, *c* = 79.6 α = 90.0, β = 113.3, γ = 90.0	*a* = 163.7, *b* = 101.0, *c* = 82.4 α = 90.0, β = 114.5, γ = 90.0
Resolution (Å)	20.0 − 1.65 (1.68 − 1.65)	19.80 − 2.50 (2.54 − 2.50)
Completeness (%)	95.2 (94.1)	98.2 (99.3)
*R*_*merge*_^a^ (%)	6.7 (81.4)	7.6 (16.5)
Reflections, Total/Unique	848,481/131,592 (6498)	190,169/41,273 (2,073)
Multiplicity	6.5 (5.7)	4.6 (4.8)
Temperature (K)	100	100
*I*/*Sigma*^b^ (*I*)	17.6 (1.4)	18.8 (6.3)
FOM^c^, Resolve (20–2.5 Å)		0.39
*R*_factor_^d^/*R*_free_^e^	0.16/0.19	
No. of atoms, protein/water	1,145/808	
Clashscore^f^, all atoms	2.01	
**Ramachandran plot**		
Favored region (%)	98.1	
Allowed region (%)	1.9	
Outliers (%)	0	
Poor rotamers (%)	0	
**Average *B* factors (Å^2^)**		
Protein	22.7	
Solvent	35.6	
**rmsds**		
Bonds (Å)	0.007	
Angles (^o^)	1.24	

*Values in parentheses are for the highest-resolution shell. FOM, figure of merit; rmsds, root-mean-square-deviations from ideal values (Engh and Huber, 1991). ^*a*^*R*_*merge*_ = å*_*hkl*_*[å*_*i*_* (| *I_*hkl,i*_-*<*I*_*hkl*_>|)]/å*_*hkl*,_*_*i*_<I*_*hkl*_*>, where *I*_*hkl,i*_ is the intensity of an individual measurement of the reflection with Miller indices *hkl* and <*I*_*hkl*_> is the mean intensity of that reflection. ^*b*^*I*/Sigma means <*I*>/σ. ^*c*^Figure of merit = ∣ *P*(α)e^*i*α^/*P*(α)∣, where *P*(α) is the phase probability distribution and α is the phase (100.0-2.75 Å). ^*d*^*R*_*factor*_ = Σ_*hkl*_∣∣*F*obs∣-∣*F*calc∣∣/Σ_*hkl*_∣*F*obs∣; where Fobs and Fcalc are the observed and calculated structure factor amplitudes, respectively, for the reflections hkl included in the refinement. ^*e*^*R*_*free*_ is the same as *R*_*factor*_ but calculated over a randomly selected fraction (10%) of the reflection data not included in the refinement. ^*f*^ Clashscore is the number of serious steric overlaps (>0.4 Å) per 1,000 atoms. The 100th percentile is the best among the structures of comparable resolution, whereas the 0th percentile is the worst.*

The traced 285 residues of zmARG form ten α-helices, seven β-strands, and a characteristic loop (α6 − α7 loop), which comprises the α/β hydrolase fold (Figure 1B). The central β-sheet formed by seven β-strands (β1-β7) is surrounded by the eight α-helices (α2-α9) and capped at one side of the β-sheet by two α-helices (α1 and α10) from one molecule and from another α-helix of a neighboring molecule (Figure 1B).

Structural similarity search using the DALI server^1^ (Holm, 2019) suggests that zmARG has a close structural relationship with other arginases, for example, arginases from *Bacillius caldovelox* (PDB ID 1CEV, bcARG, Z-score 27.9, RMSD 2.6 Å, sequence identity 19%) (Bewley et al., 1999), *Trypanosoma brucei* (4RHI, tbARG, 17.4, 3.7 Å, 15%) (Hai et al., 2015), *Rattus norvegicus* (1HQF, rnARG, 26.6, 3.0 Å, 18%) (Cox et al., 2001), and *Homo sapiens* (2ZAV, hsARG, 26.6, 2.8 Å, 18%) (Di Costanzo et al., 2007b). Although the sequence identity between the zmARG and the related proteins are relatively low, the overall architecture of zmARG is similar with others, as suggested with high *Z*-score values and reasonable rmsd values. Interestingly, the last helix (α10-helix) of zmARG that is not conserved well in other homologous structures orients the neighboring molecule (Figure 1B). In addition, the loop between the β6-strand and the α9-helix is relatively longer than other homologs, which has several acidic residues and forms a negatively charged patch (Figure 1C and Supplementary Figure S2).

### Active Site of zmARG

Arginases catalyze the arginine hydrolase reaction aided by two manganese ions that are coordinated with the conserved sequence motifs within the active site (Ouzounis and Kyrpides, 1994; Perozich et al., 1998).

**FIGURE 2:**
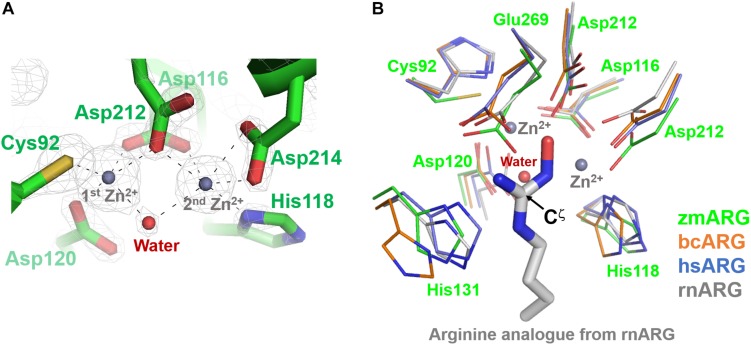
Active site of zmARG. **(A)** *2Fo–Fc* electron density map (contoured at 3.0 σ level) in the active site. Interaction between the zinc ions and protein atoms are indicated with black-dotted lines. Protein side chains are drawn with stick models. Two bound zinc ions and a water molecule bridging two zinc ions are displayed with gray and red spheres, respectively. **(B)** Comparison of the zmARG site with those of bcARG, hsARG, and rnARG. Active site residues of thin stick models are differentiated by colors. The substrate analog N-omega-hydroxyl-l-arginine bound to rnARG is shown as thick stick models. The zinc ions (gray) and water molecule (red) are drawn by spheres.

The refined zmARG structure has shown two strong spherical electron densities in the pocket of each α/β hydrolase fold, which were modeled as zinc ions (Figure 2), based on the X-ray absorption near edge structure (XANES) and a fluorescence scan (Supplementary Figure S3). The modeled zinc ions might be derived from the culture media, protein purification buffers, or crystallizing solutions. The deep pocket with the two metal-binding sites of zmARG is lined with residues of the arginase-forming conserved sequence motifs of ^89^GGDCL^93^, ^116^DSHPD^120^, and ^210^HIDLDVLDP^218^ (Supplementary Figure S2 and Figure 2).

The two modeled zinc ions shares the Oδ of Asp116 (bidentate), Asp212, and one metal-bridging water molecule (Figure 2A). The first zinc ion is coordinated with five atoms of Sγ of Cys92, three Oδs of Asp116, Asp120, and Asp212, and the metal-bridging water molecule, which forms a distorted trigonal bipyramid configuration. On the other hand, the second zinc ion is hexa-coordinated with four Oδ atoms of Asp116, Asp212, and Asp214 (bidentate), Nε atom of His118, and the metal-bridging water molecule, and forms an octahedral geometry (Figure 2A).

### Oligomeric State of zmARG

While most of the mammalian arginases form a trimeric structure, bacterial ones form a monomeric or hexameric structure (Carvajal et al., 1977; Aguirre and Kasche, 1983; Jenkinson et al., 1996; Kanyo et al., 1996; Bewley et al., 1999).

**FIGURE 3:**
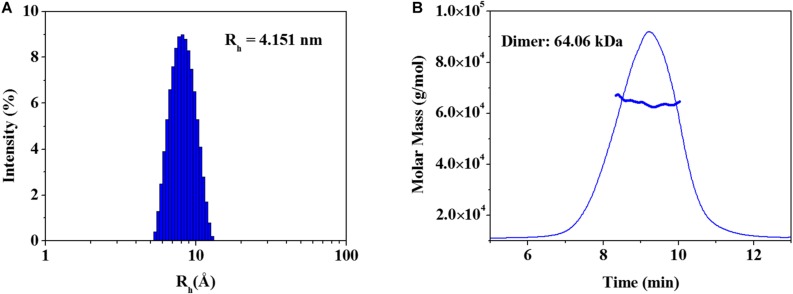
Analysis of oligomeric states of the recombinant zmARG. **(A)** Dynamic light scattering (DLS). Intensity-weighted hydrodynamic radius (R_h_) distribution of zmARG protein in solution was plotted. The protein concentration was 3 mg/ml in 20 mM Tris–HCl (pH 7.5) with 150 mM NaCl. **(B)** Asymmetrical flow field-flow fractionation coupled with multi-angle light scattering (AF4-MALS) for zmARG protein in solution. The thick line represents the determined molecular weight by the Zimm model.

The elucidated zmARG structure has four molecules in the asymmetric unit. Among the four molecules, two molecules form a dimeric structure (Figure 1B). The other two molecules interact with symmetrically related molecules in the crystal system and form the same dimeric structure as that in the asymmetric unit. However, zmARG does not appear to form a higher oligomeric state in the crystal structure because of a limited number of non-specific interactions among the nearby dimeric proteins. To further characterize the oligomeric state of zmARG in solution, we carried out a dynamic light scattering (DLS) analysis, which showed one major peak for distribution of particles with a mean hydrodynamic radius of 4.151 nm (Figure 3A). The estimated protein size from DLS almost coincides the diameter of ∼80 Å along the longest axis in the current dimeric zmARG structure. Asymmetrical flow field-flow fractionation with multi-angle light scattering suggested a calculated molecular weight of 64 kDa, which corresponds to the expected dimeric molecular weight of 64 kDa of zmARG (Figure 3B). These experimental data strongly support that zmARG retains its dimeric state in solution.

In forming a dimeric zmARG structure, two molecules interact with each other in a C-terminus tail-to-tail manner (Figure 1B). Notably, the C-terminal tail including the α10-helix (Trp273-Glu284) of each protomer ptotrudes outside from the globular α/β hydrolase fold and interacts with the open hydrophobic pocket on the surface formed by the two α-helices (α1 and α9) and the central β-sheet of the neighboring molecule (Figure 1B and Supplementary Figure S4). In addition, a few of the direct polar interactions (Asp274 and Glu284) except the water-mediated polar interactions contribute to form a stable dimeric zmARG structure.

Collectively, the observed structural features and biophysical results strongly indicate that zmARG exists as a dimeric state in solution, whose dimeric structure is most likely similar to that observed in the current crystal structure.

## Discussion

### zmARG Is Also a Manganese Ion-Dependent Arginase

All the reported arginases, whether they originate from mammals or bacteria, are Mn ion-dependent enzymes (Bewley et al., 1999; Cox et al., 2001; Di Costanzo et al., 2007a; Hai et al., 2015).

Our high-resolution crystal structure of zmARG has shown two strong spherical electron densities within a typical arginase fold (Figure 2A). Furthermore, XANES and fluorescence scanning suggested the presence of zinc ions in the crystal (Supplementary Figure S3). Therefore, we arbitrarily positioned two zinc ions within these electron densities. However, the purified recombinant zmARG protein has an arginine-hydrolyzing activity only in the presence of manganese ions with a negligible cleaved product with zinc ions (right in Figure 1A), when the arbitrarily incorporated metal ions were removed with EDTA before assays. Notably, many metal ion-binding proteins are often purified with intrinsic zinc ions when their recombinant proteins are expressed in *E. coli* (Foster et al., 2014). Based on the assays with deprivation of the metal ions taken from the expression host and loading metal ions into the active site (right in Figure 1A), it can be argued that zmARG is also a manganese ion-dependent arginase like other arginases.

### zmARG Is a Dimer in Solution

The reported arginase structures and biochemical characterization demonstrate their various oligomeric states. Mammalian arginases, for example, human arginase I (Di Costanzo et al., 2007a), II (Cama et al., 2003), and rat liver arginase (Kanyo et al., 1992), commonly form a trimeric structure. On the other hand, bacterial arginases are not uniform in their oligomeric states, for example, a monomeric arginase from *Helicobacter pylori*, a monomeric and dimeric structure of *Entamoeba histolytica*, and a hexameric arginase formed by a dimer of trimers from *Bacillus caldovelox* (Bewley et al., 1999; Malik et al., 2018). Interestingly, the key structural feature of a higher oligomeric state is characterized by the presence of a unique S-shaped oligomerization motif (S-tail) at the C-terminus of the protein that mediates interaction among monomers (Lavulo et al., 2001). Analysis of mutations in the S-tail motif suggests that the extreme C-terminal conformation determines their respective oligomeric states (Lavulo et al., 2001) (Supplementary Figure S5).

The revealed zmARG structure implies that zmARG form a dimeric structure by exchanging the terminal α-helix that swings over the globular domain of the neighboring subunit and forms many hydrophobic interactions (Figure 1B and Supplementary Figure S4). Notably, this kind of interaction in forming dimeric proteins, namely, swapping of one secondary structure element of one protomer with the globular part of the neighboring subunit, is also observed in other dimerzing proteins of the alcohol dehydrogenase zmADH from *Z. mobilis* (Moon et al., 2011) and the nitroreductase Ydja from *E.coli.* (Choi et al., 2008). Since our biophysical assays have also shown that zmARG exists as a dimeric form in solution (Figure 3), zmARG functions as a dimer mediated with an S-tail-like helix, a structural feature typically observed in the higher oligomeric arginases.

### Metal Coordination of zmARG Is Different From Other Arginases

The three conserved sequence motifs of GGDHS, DAHXD, and SXDXDXDP (X is any amino acid) in arginases are important for coordination of the catalytic bimetallic cluster. The histidine of GGDHS motif ligands and stabilizes the first manganese ion, whereas the GG with an unusual cis−peptide bond may be essential for maintaining a catalytically competent structure for optimum activity (Kanyo et al., 1992; Bewley et al., 1999; Srivastava et al., 2011). The side chains of the first aspartate and the histidine residue of the DAHXD motif chelate the second metal ion. In the SXDXDXDP motif, the first aspartate chelate both metal ions and the second aspartate acts as a second metal ligand.

zmARG also has three sequence motifs of GGDCL, DSHPD, and HIDLDVLDP (Figure 1A). However, these sequence motifs are slightly different from those of other compared arginases. Notably, the histidine residue of the GGDHS motif liganding the first metal ion in other arginases (Kanyo et al., 1992; Bewley et al., 1999; Srivastava et al., 2011) is replaced with the cysteine residue (Figure 2B). Furthermore, the first zinc ion of zmARG forms a trigonal bipyramid by five ligands while the second one is octahedrally coordinated by six ligands (Figure 2A), which is different from two metal ions of an octahedral geometry in other arginases (Figure 2B). The first metal ion-binding site shows a diversity in metal coordinating ligands as well as its geometry, for example, two water molecules in bcARG and hsARG and a square pyramidal geometry in rnARG (Kanyo et al., 1996; Bewley et al., 1999; Di Costanzo et al., 2007a). Therefore, this unusual trigonal bipyramid geometry of the first metal ion in zmARG may not be an exceptional case and have another geometry during the catalysis as suggested in rnARG (Christianson, 2005).

To gain insights into molecular interactions between the arginine substrate and the zmARG, we tried to get a zmARG structure in complex with arginine, but failed until now. Therefore, we superposed our zmARG structure and the ARG structures in complex with arginine analog (Bewley et al., 1999; Cox et al., 2001; Di Costanzo et al., 2007a; Hai et al., 2015), which suggests a similar binding pose of the substrate in the active of zmARG to those in other arginases (Figure 2B). These structural similarities at the active site, despite the different metal-coordination geometry, suggest that zmARG may share the same substrate-binding mode and common catalytic mechanism with other arginases.

Interestingly, the superposed structures highlight a flexibility of the β6 − α9 loop with respect to the core site (Supplementary Figure S5). Sequences of the β6 − α9 loop are not conserved well and zmARG has several inserted residues between the end of the SXDXDXDP motif and the capping residue of the α9 helix, compared with other arginases (Supplementary Figures S2, S5). Furthermore, this β6 − α9 loop has many acidic residues that form a negatively charged patch at the entrance part of the active site, suggesting its another conformation upon substrate binding presumably by interacting with the positively charged β4-α4 loop by conserved basic residues (Figure 1C and Supplementary Figure S2).

## Conclusion

In this study, we have shown that zmARG catalyzed the arginine-hydrolyzing activity in a manganese ion-dependent manner and the dimeric state is formed by a novel helix-swapping manner. *Z. mobilis* ZM4 is a representative ethanologenic organism with ethanol tolerance. Expression of the zmARG (ZMO0432) was significantly increased at a high ethanol-stressed condition (Yang et al., 2013). It is not clear how zmARG is related with the alcohol tolerance of the organism. However, it is worthwhile to mention that nitrogen metabolism plays a critical role in the alcohol tolerance by enabling ethanol-stressed cells to overcome the carbon metabolism inhibition under the ethanol shock (Yang et al., 2012). Since the arginine degradation by zmARG contributes to the vital nitrogen metabolism (Jenkinson et al., 1996), further study will clarify the probable direct or indirect relationship of zmARG with alcohol tolerance of *Z. mobilis* ZM4.

## Data Availability Statement

The datasets generated for this study can be found in the Protein Data Bank 6KSY.

## Author Contributions

S-YP and J-SK designed the study. All authors performed the experiments, analyzed data, and wrote the manuscript.

## Conflict of Interest

The authors declare that the research was conducted in the absence of any commercial or financial relationships that could be construed as a potential conflict of interest.

## Footnotes

1 http://ekhidna2.biocenter.helsinki.fi/dali/

## Supplementary Material

FIGURE S1 Analysis of steady-state zmARG kinetics. Data points show the steady-state velocity for different initial substrate concentrations. The solid line represents fit of the Michaelis–Menten equation **(left)** and Lineweaver-Burk plot **(right)**. 31 μM zmARG was used for enzyme kinetics.

FIGURE S2 Sequence alignment of an arginase (zmARG) from *Zymomonas mobilis* ZM4 and homologs from *Bacillius caldovelox* (bcARG), *Trypanosoma brucei* (tbARG), and *Homo sapiens* (hsARG). The cylinders and arrows above the aligned sequences represent α-helices and β-strands, respectively, in the order of their appearances. The numbering at every 10th residue follows the amino-acid sequence of zmARG. Residues that are identical across all sequences are marked “^∗^”. Conserved residues are marked with “:” or “.”. The conserved sequence motifs suggested for catalysis and metal ion-coordination are boxed by blue and green colors, respectively. The 10th helix that is key element for the formation of a dimeric structure is marked with a red box.

FIGURE S3 Fluorescence scan at 10 Kev (left) and X-ray absorption near-edge structure (XANES) measurement (right). The X-ray fluorescence and the absorption scan were performed at the zinc peak.

FIGURE S4 Dimeric interface of zmARG. Two subunits are differentiated by colors. Some protein residues and zinc ions are drawn by thick stick models and sphere models, respectively, (gray).

FIGURE S5 Comparison of the dimeric zmARG with a trimeric hsARG structure. The superimposed zmARG (green/cyan) and hsARG (blue) structures of two views are displayed by coils with the zmARG α10-helix of a ribbon.

TABLE S1 Comparison of kinetic parameters for zmARG and other arginases.
