# Supplementary figures and images for: Characterization of a Dimeric Arginase From Zymomonas mobilis ZM4
Source: Front Microbiol. 2019 Nov 26;10:2755. doi: 10.3389/fmicb.2019.02755 (PMC6988801; doi:10.3389/fmicb.2019.02755)

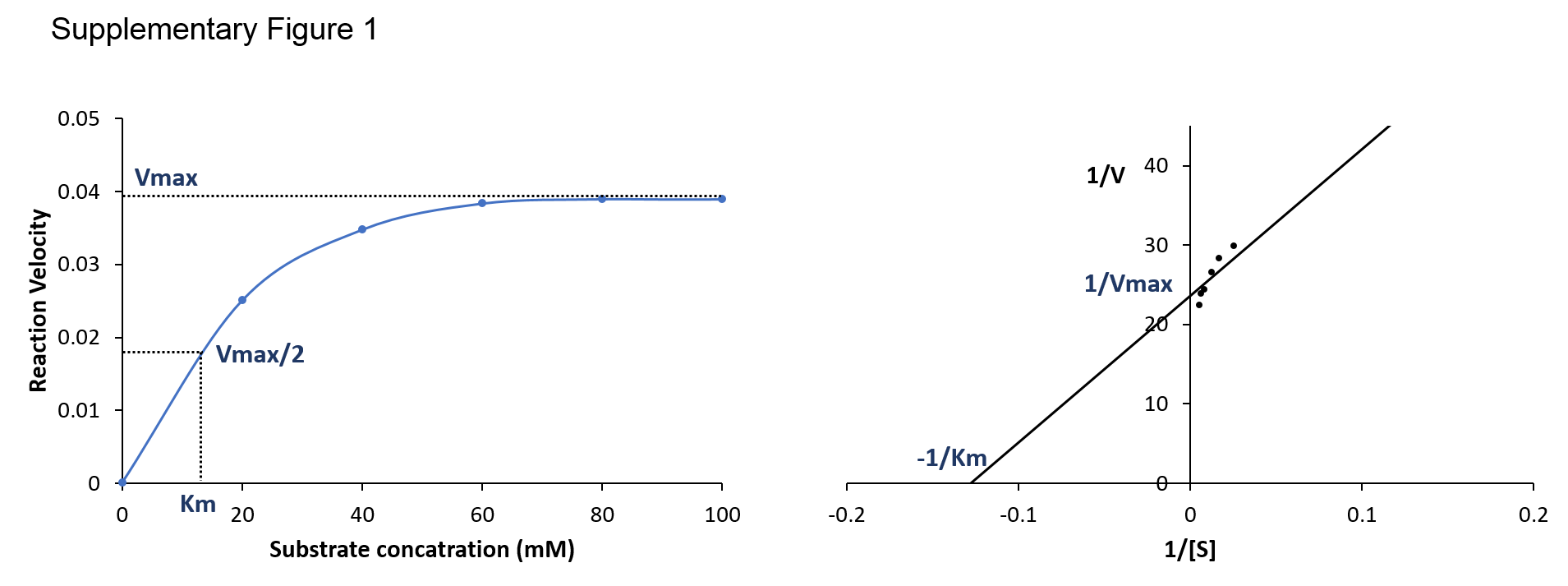

Supplement: FIGURE S1 — Analysis of steady-state zmARG kinetics. Data points show the steady-state velocity for different initial substrate concentrations. The solid line represents fit of the Michaelis–Menten equation (left) and Lineweaver-Burk plot (right). 31 μM zmARG was used for enzyme kinetics. [file Image_1.TIF]

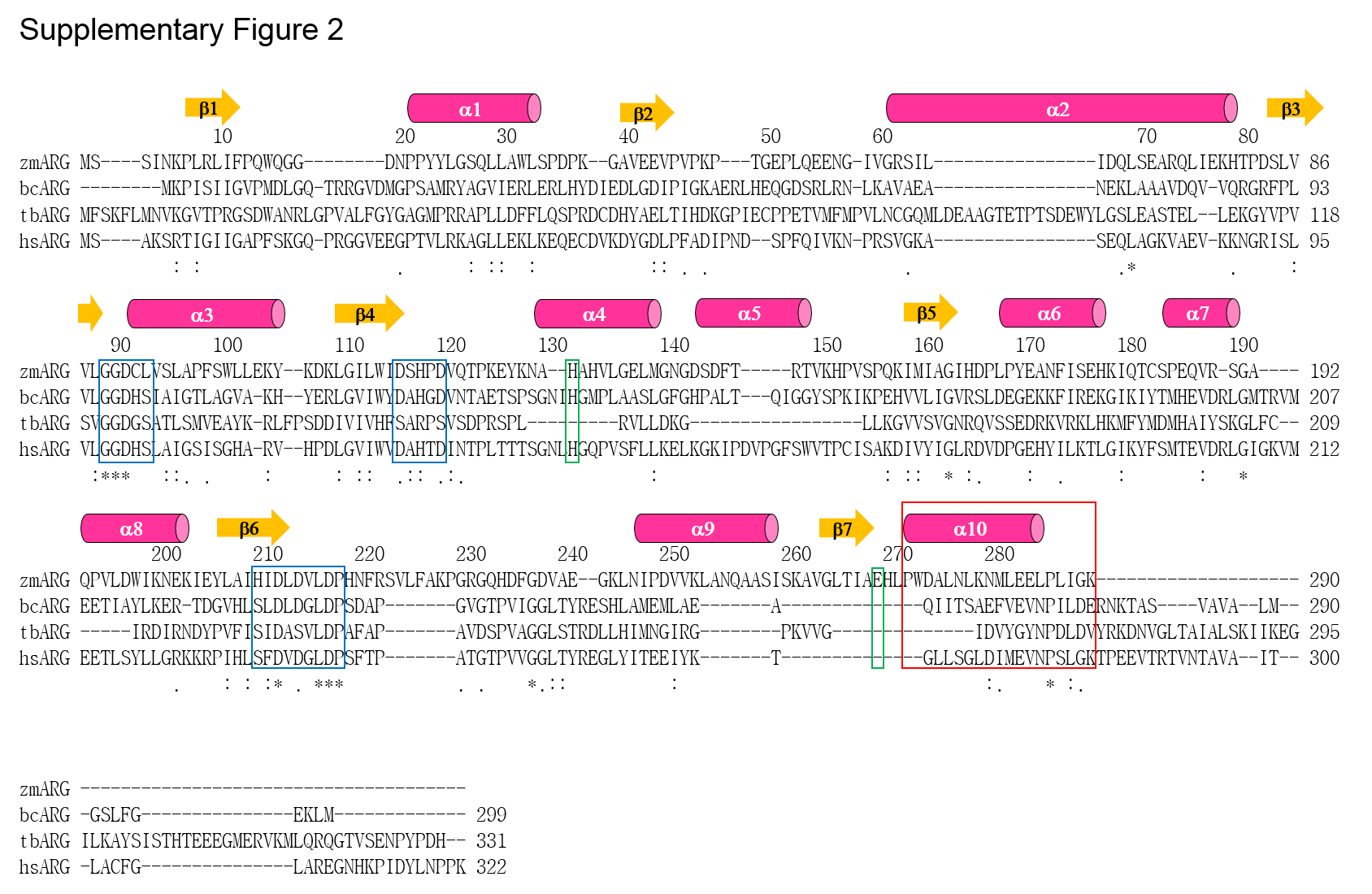

Supplement: FIGURE S2 — Sequence alignment of an arginase (zmARG) from Zymomonas mobilis ZM4 and homologs from Bacillius caldovelox (bcARG), Trypanosoma brucei (tbARG), and Homo sapiens (hsARG). The cylinders and arrows above the aligned sequences represent α-helices and β-strands, respectively, in the order of their appearances. The numbering at every 10th residue follows the amino-acid sequence of zmARG. Residues that are identical across all sequences are marked “∗”. Conserved residues are marked with “:” or “.”. The conserved sequence motifs suggested for catalysis and metal ion-coordination are boxed by blue and green colors, respectively. The 10th helix that is key element for the formation of a dimeric structure is marked with a red box. [file Image_2.TIF]

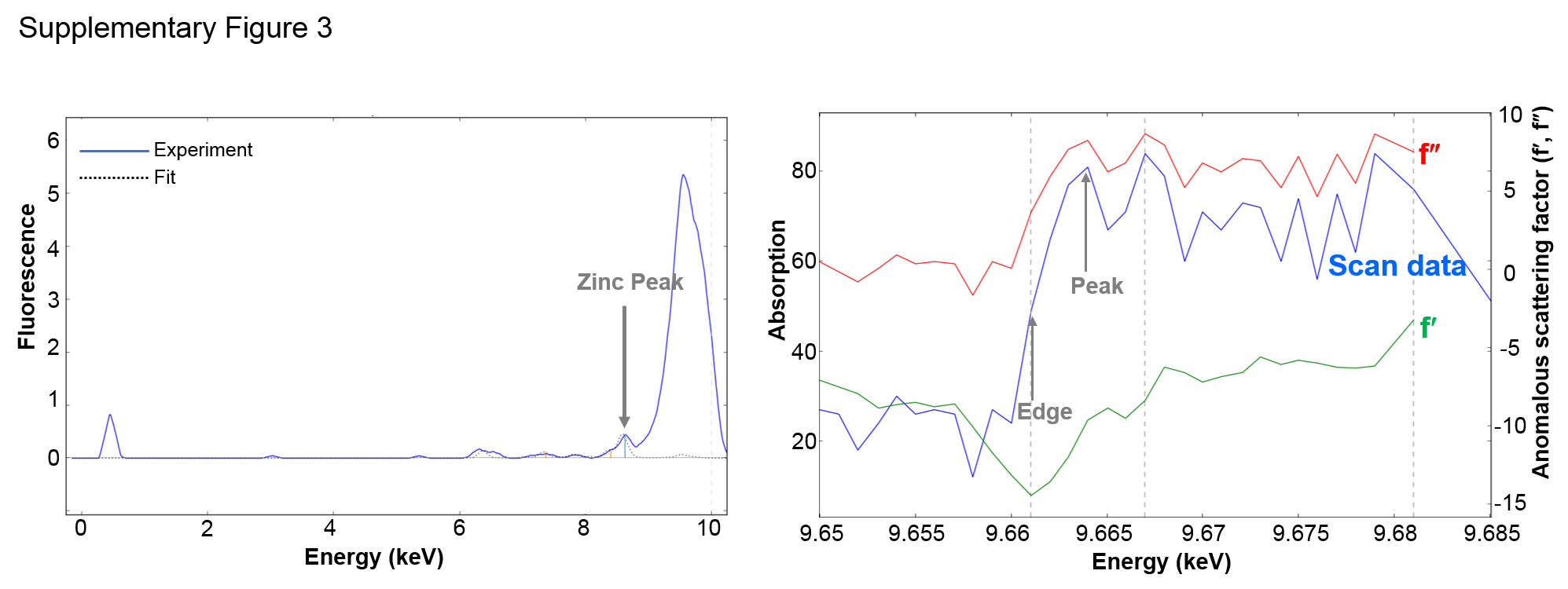

Supplement: FIGURE S3 — Fluorescence scan at 10 Kev (left) and X-ray absorption near-edge structure (XANES) measurement (right). The X-ray fluorescence and the absorption scan were performed at the zinc peak. [file Image_3.TIF]

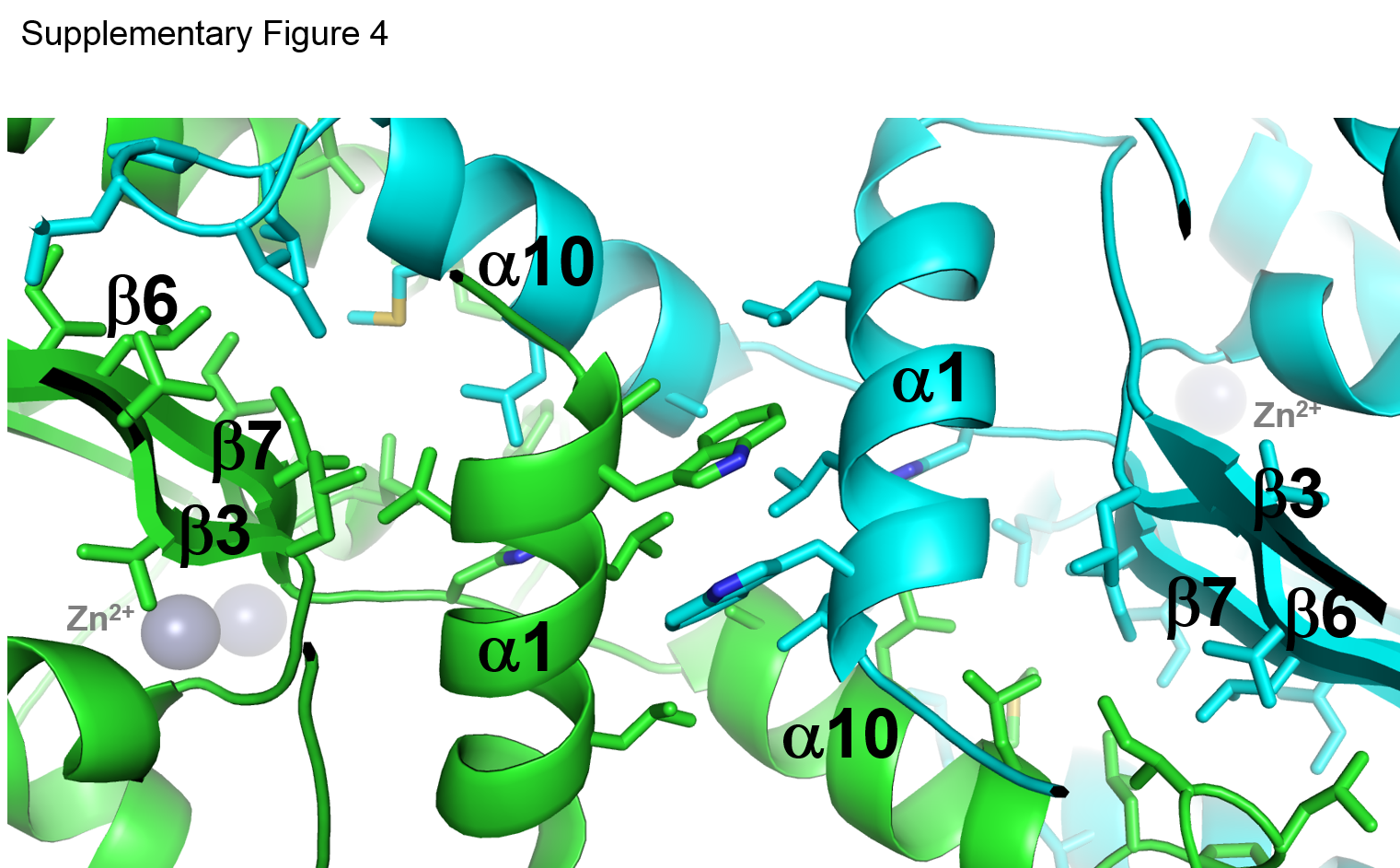

Supplement: FIGURE S4 — Dimeric interface of zmARG. Two subunits are differentiated by colors. Some protein residues and zinc ions are drawn by thick stick models and sphere models, respectively, (gray). [file Image_4.TIF]

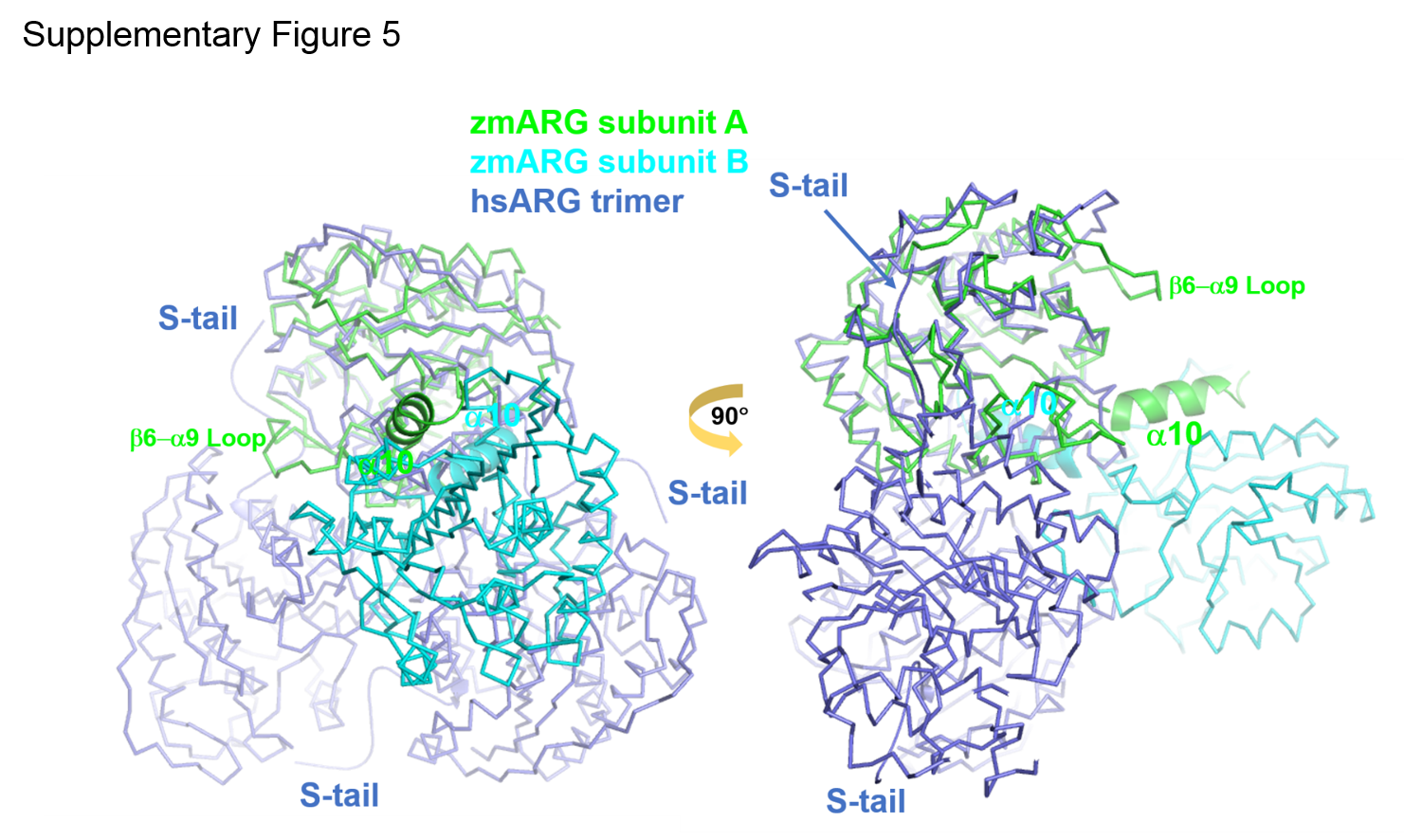

Supplement: FIGURE S5 — Comparison of the dimeric zmARG with a trimeric hsARG structure. The superimposed zmARG (green/cyan) and hsARG (blue) structures of two views are displayed by coils with the zmARG α10-helix of a ribbon. [file Image_5.TIF]
