# Supplementary material for: Characterization of a Dimeric Arginase From Zymomonas mobilis ZM4
Source: Front Microbiol. 2019 Nov 26;10:2755. doi: 10.3389/fmicb.2019.02755 (PMC6988801; doi:10.3389/fmicb.2019.02755)
Supplement: TABLE S1 — Comparison of kinetic parameters for zmARG and other arginases. [file Table_1.DOCX]

Supplementary Table 1. Comparison of kinetic parameters for zmARG and other arginases

| Parameter | zmARG | hsARG | rnARG | bcARG |
| --- | --- | --- | --- | --- |
| Km | 6.8 mM | 1.5 mM | 4.4 mM | 3.4 mM |
| Kcat | 302/s | 190/s | 70/s | 700/s |
| η = Kcat/Km | 44,000/s/M | 127,000/s/M | 16,000/s/M | 206,000/s/M |
